# Femur Osteomyelitis and Associated Fracture as an Initial Presentation of Aortoenteric Fistula

**DOI:** 10.1055/s-0042-1757794

**Published:** 2022-12-20

**Authors:** Emmanouil Barmparessos, Petros Chatzigakis, Vasileios Katsikas, Andreas Zevlas, Dimitrios Samaras

**Affiliations:** 1 Department of Vascular Surgery, General Hospital of Athens “Georgios Gennimatas,” Athens, Greece; 2 1st Surgical Department, General Hospital of Athens “Georgios Gennimatas,” Athens, Greece; 3 Department of Orthopedic Surgery and Traumatology, General Hospital of Piraeus “Tzaneio,” Piraeus, Greece

**Keywords:** osteomyelitis, pathological fracture, aortobifemoral bypass graft infection, aortoenteric fistula

## Abstract

Aortoenteric fistula is a rare condition. Atypical presentations may cause significant management delays. We present the case of a 64-year-old male who experienced a pathological femoral fracture as an initial presentation of an underlying aortoenteric fistula. The aortoenteric fistula, possibly related to a poor graft tunneling technique, induced femur osteomyelitis and the associated pathological fracture.

## Introduction

Aortoenteric fistula is a rare condition. It presents with the high complexity of management and a high mortality rate. 1 We present an unusual case wherein a pathological femoral fracture was the initial manifestation of the underlying aortoenteric fistula. Informed consent was obtained from the patient's next of kin before publishing his images and history; therefore, approval by the institutional review board was waived.

## Case Presentation

A 64-year-old male patient underwent an aortobifemoral bypass, using a polyethylene terephthalate (PET) graft, in another institution owing to lifestyle limiting claudication.

Seven months after his primary aortic surgery, a diaphyseal femoral fracture occurred while he was resting. Given the absence of trauma or stress, the fracture was considered pathological.

**Fig. 1:**
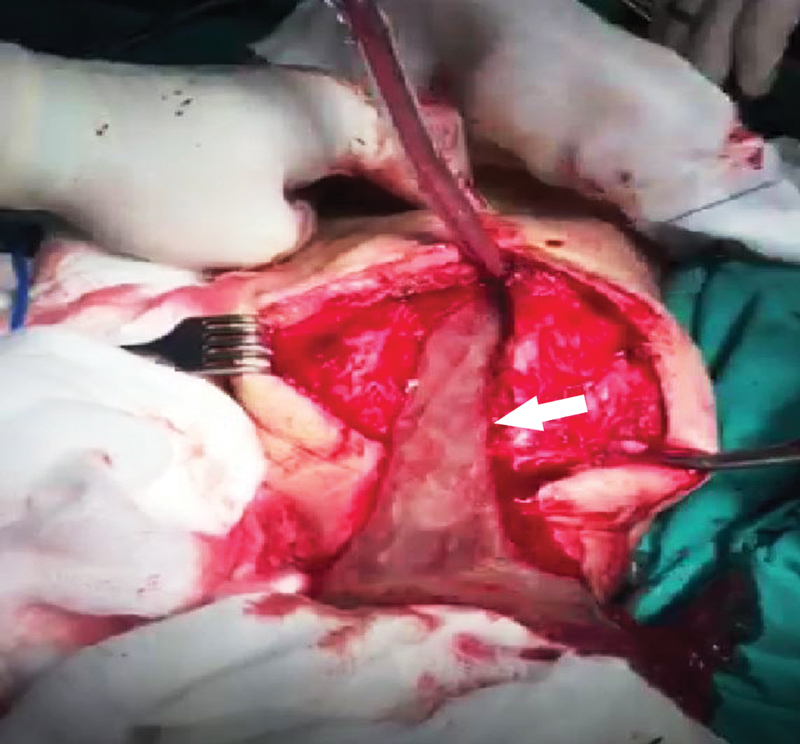
Intraoperative image of the purulent exudation around the femur (shown by white arrow) when external osteosynthesis was performed.

To treat his femoral fracture, external osteosynthesis was performed along with extensive purification and drainage because of intraoperative purulent exudation around his femur ( Fig. 1 ).

The intraoperative periosteal inflammatory tissue culture revealed two strains of oral gram-positive anaerobic cocci ( *Fusobacterium nucleatum* and *Parvimonas micra* ).

Considering the origin of the infection, computed tomography (CT) scan was performed but was reported without any pathological findings regarding abdominal or other system infection.

**Fig. 2:**
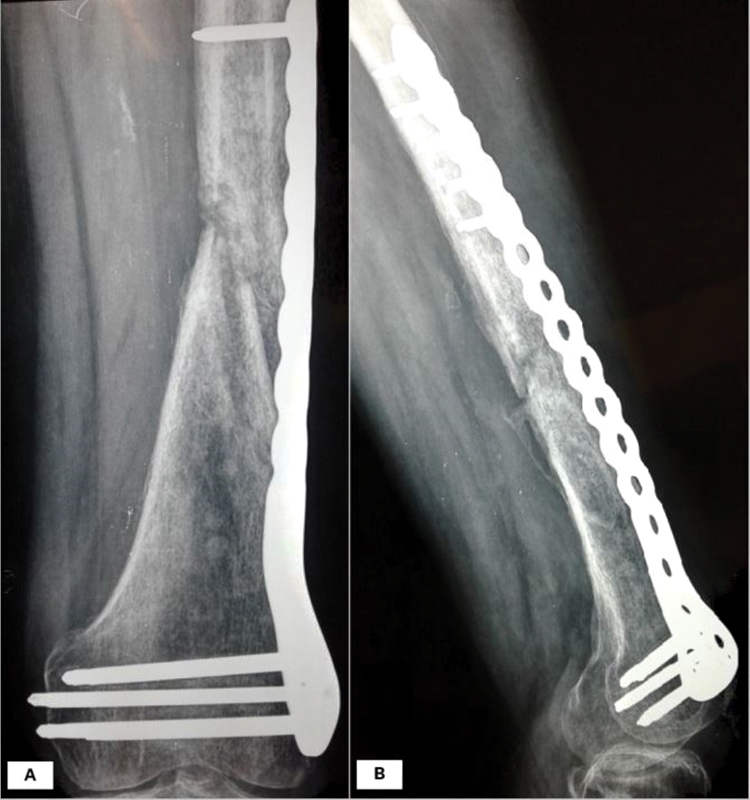
Radiographic image of the fracture following implantation of antibiotic-impregnated cement and distal femur plating system.

Three months after orthopedic surgery, while being afebrile, his external osteosynthesis was removed. After repeating a debridement, antibiotic-impregnated cement and a distal femur plating system were introduced ( Fig. 2 ).

Despite initial improvement, a localized mass appeared in his left popliteal fossa in the following weeks. The mass was irrigated and a culture isolated *Escherichia coli* , *Enterococcus faecalis* , and *Proteus mirabilis* .

Owing to fever along with extensive edema in his left thigh and elevated inflammatory blood markers (white blood cell [WBC] count: 12.3 × 109/L, erythrocyte sedimentation rate [ESR]: 78 mm/h, C-reactive protein [CRP]: 300 mg/L)], the patient was readmitted to the orthopaedic service with a diagnosis of osteomyelitis. Radiographic images confirmed the diagnosis; however, blood cultures were negative. Despite an extended antibiotic regimen, the swelling in his left popliteal fossa relapsed. This finding was accompanied by an additional palpable mass in his left inguinal ligament. He was then referred to our vascular surgery department.

**Fig. 3:**
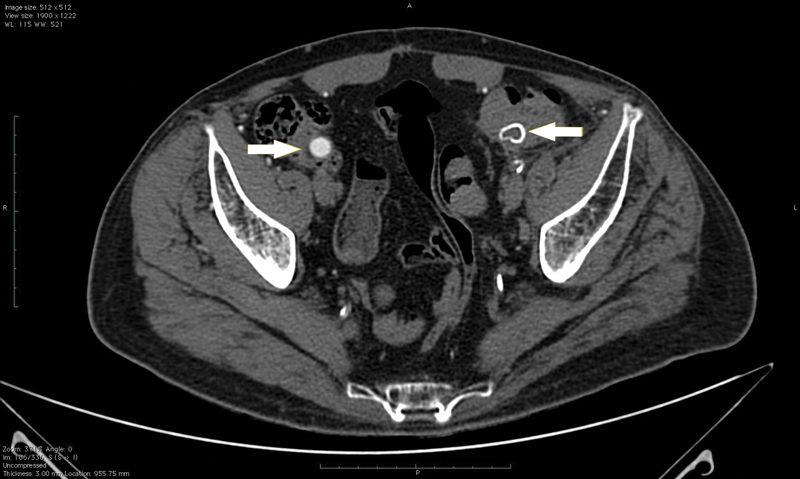
Preoperative computed tomography scan. Axial image indicating that the thrombosed left and right limbs of the graft (shown by white arrows) have an intraluminal course through the sigmoid and cecum, respectively.

**Fig. 4:**
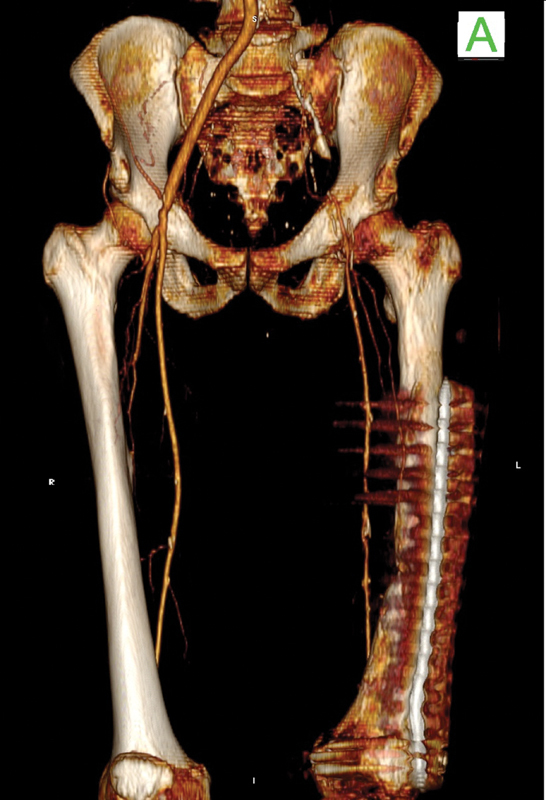
Preoperative three-dimensional computed tomography scan reconstruction. The anterior view shows the thrombosed left limb of the polyethylene terephthalate graft with sufficient ipsilateral distal perfusion.

The patient underwent a new CT angiography scan that revealed a thrombosed left limb of his aortobifemoral PET graft, with the impression that the thrombosed segment, as well as the right limb of the aortic graft, took an intraluminal course at the proximal end level of his sigmoid colon and cecum, respectively ( Fig. 3 ). Moreover, the scan revealed fluid and gas accumulation surrounding his left femoral anastomosis and sufficient distal perfusion to the left leg despite the thrombosed left limb of the graft ( Fig. 4 ).

Inflammatory markers normalized (WBC: 7.1 × 109/L, ESR: 35 mm/h, CRP: 20 mg/L). A course of intravenous antibiotics (ceftazidime/avibactam and tigecycline) was instituted, and a transperitoneal approach exposed the aortic graft under general anesthesia. In addition to excessive fluid accumulation around the aortic graft, it was found that the right and left iliac limbs of his PET graft transversed the cecum and sigmoid colon, respectively.

Following total explantation of his aortobifemoral graft, accompanied by wide debridement and retroperitoneal irrigation, an appendectomy and sigmoidectomy were performed.

**Fig. 5:**
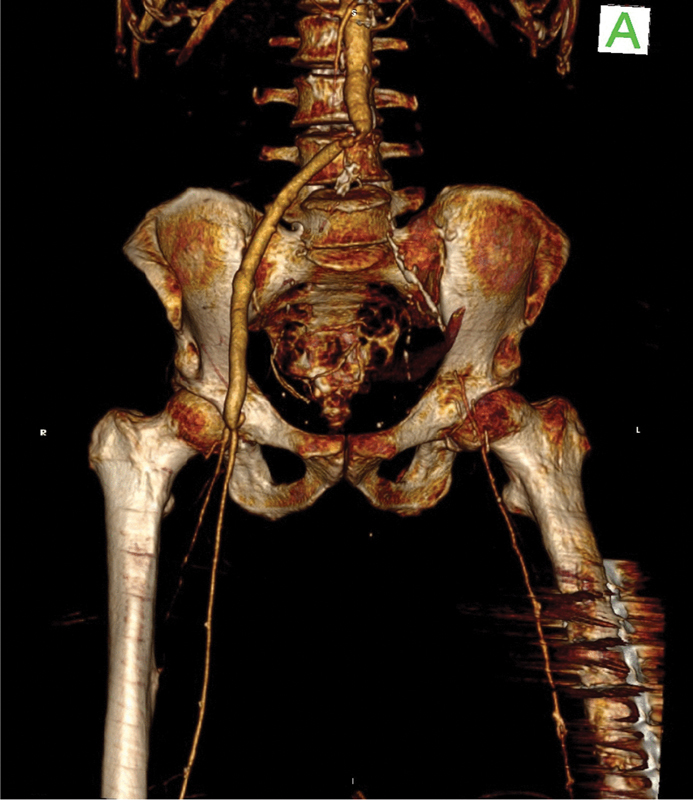
Postoperative three-dimensional computed tomography scan reconstruction. The anterior view shows the patent aortofemoral bypass and sufficient perfusion to the left leg owing to collateral circulation.

Considering the extensive inflammation in his left inguinal area, and the absence of ischemic symptoms despite the thrombosed left limb of the PET graft, we proceeded with a bailout procedure by creating a composite graft consisting of his harvested right femoral vein and a short segment of tubular silver-impregnated PET graft. The length of the autologous graft alone was inadequate ( Fig. 5 ). The tubular silver-impregnated PET graft was anastomosed proximally to the aortic stump and distally to the autologous venous graft which was consecutively anastomosed to the right femoral bifurcation.

Despite initial postoperative improvement, sigmoid colon necrosis was observed on day 3 and Hartmann's colectomy procedure was thus performed. Owing to the freedom of the aortofemoral bypass from the above pathology, no reintervention to the graft was attempted. Eventually, the patient died of sepsis because of peritonitis after a prolonged stay (16 days) in the intensive care unit.

## Discussion

Aortic graft infection is a rare condition, with a reported incidence of 1 to 4%, while aortoenteric fistula has an incidence of 0.4 to 2%. 1 However, aortic graft infection and aortoenteric fistula pose a great challenge. Following surgical treatment for aortic graft infection, mortality is 18 to 30%, an even higher when accompanied by aortoenteric fistula, reaching an average of 30 to 40%. 1

The clinical presentation of aortic graft infection varies, depending on the time of symptom onset after the primary aortic surgery. 2 In early onset of aortic graft infection, symptoms appear within the 4 months following aortic surgery. In these cases, patients present with signs of systemic infection such as fever and elevation of inflammatory markers; patients may complain of local signs of wound infection.

Conversely, in late-onset cases, symptoms are manifested from 4 months up to 10 years after the primary aortic surgery and are associated with less virulent but more fastidious microorganisms. In these cases, patients complain of nonspecific symptoms such as malaise, fatigue, weight loss, intermittent fever, intermittent claudication, back pain, localized inguinal mass, and gastrointestinal bleeding when aortoenteric fistula is also present. 2 Sporadically, the extension of aortic graft infection to the vertebral fascia by the contiguous infectious process may cause spondylitis. 3 Another rare presentation of aortic graft infection is hypertrophic osteoarthropathy (periosteal new bone formation, digital clubbing, and synovitis) with almost 30 cases reported in the literature. 4

In our case, the malposition of the iliac limbs of the graft tangentially with the cecum and sigmoid colon gave rise to the aortoenteric fistula during the tunneling.

Based on the literature, there have already been reports of iatrogenic perforation of the colon during tunneling of an aortobifemoral graft; however, our patient exhibited a unique presentation. 5 To the best of our knowledge, there have been no other similar cases of pathological fracture as an initial presentation of underlying aortoenteric fistula in the English literature. In our case, the aortoenteric fistula led to periaortic inflammation that infiltrated the surrounding tissues. The expansion of the contiguous infectious process to the femur induced osteomyelitis and a pathological femoral fracture. The atypical presentation caused a significant delay in diagnosis that affected his outcome.

In conclusion, poor graft tunneling technique caused an aortoenteric fistula that induced femoral osteomyelitis and an associated femoral fracture. Regarding the underlying pathology, aortic graft infection may exhibit an unusual presentation; therefore, vigilance regarding aortic surgery complications is crucial.

## References

[1] Esvs Guidelines Committee Chakfé N Diener H Lejay A Editor's choice–European Society for Vascular Surgery (ESVS) 2020 clinical practice guidelines on the management of vascular graft and endograft infections Eur J Vasc Endovasc Surg 2020 59 03 339 384 32035742 10.1016/j.ejvs.2019.10.016

[2] FitzGerald S F Kelly C Humphreys H Diagnosis and treatment of prosthetic aortic graft infections: confusion and inconsistency in the absence of evidence or consensus J Antimicrob Chemother 2005 56 06 996 999 16269550 10.1093/jac/dki382

[3] Orton D F LeVeen R F Saigh J A Aortic prosthetic graft infections: radiologic manifestations and implications for management Radiographics 2000 20 04 977 993 10903688 10.1148/radiographics.20.4.g00jl12977

[4] Alonso-Bartolomé P Martínez-Taboada V M Pina T Blanco R Rodriguez-Valverde V Hypertrophic osteoarthropathy secondary to vascular prosthesis infection: report of 3 cases and review of the literature Medicine (Baltimore) 2006 85 03 191 16721260 10.1097/01.md.0000224714.27508.8b

[5] Blank J J Rothstein A E Lee C J Aortic graft infection secondary to iatrogenic transcolonic graft malposition Vasc Endovascular Surg 2018 52 05 386 390 29554857 10.1177/1538574418764037

